# The effect of recanalization of a chronic total coronary occlusion on P-wave dispersion

**DOI:** 10.34172/jcvtr.2021.38

**Published:** 2021-08-25

**Authors:** Aydın Rodi Tosu, Muhsin Kalyoncuoğlu, Halil İbrahim Biter, Sinem Çakal, Beytullah Çakal, Tufan Çınar, Erdal Belen, Mehmet Mustafa Can

**Affiliations:** ^1^Health Sciences University, Haseki Training and Research Hospital, Department of Cardiology, Istanbul, Turkey; ^2^Istanbul Medipol University, Faculty of Medicine, Department of Cardiology, Istanbul, Turkey; ^3^Health Sciences University, Sultan II. Abdulhamid Han Training and Research Hospital, Department of Cardiology, Istanbul, Turkey

**Keywords:** Chronic Total Occlusion, Percutaneous Coronary, Intervention, P-Wave, P-Wave Dispersion

## Abstract

***Introduction:*** P-wave dispersion (PWD) obtained from the standard 12-lead electrocardiography (ECG) is considered to reflect the homogeneity of the atrial electrical activity. The aim of this investigation was to evaluate the effect of percutaneous chronic total occlusion (CTO) revascularization on the parameters of P wave duration and PWD on ECG in cases before and after procedure at 12^th^ months.

***Methods:*** We analyzed 90 consecutive CTO cases who were on sinus rhythm and underwent percutaneous coronary intervention (PCI). P-wave maximum (P_-max_) and P-wave minimum (P_-min_), P-wave time, and PWD were determined before and twelve months after the CTO intervention. The study population was categorized into two groups as successful and unsuccessful CTO PCI groups.

***Results:*** The CTO PCI was successful in 71% of cases (n=64) and it was unsuccessful in 29% of cases (n=26). Both groups, except for age and hypertension, were similar in terms of demographic and clinical aspects. CRP levels were significantly elevated in the unsuccessful CTO PCI group. Pre-PCI ECG parameters showed no significant difference. Irrespective of the target vessel revascularization, we observed that PWD and P_-max_ values were significantly lower in the 12^th^ months follow-up. In all Rentrop classes, PWD values were significantly decreased at 12^th^ months follow-up in comparison to the pre-CTO PCI values.

***Conclusion:*** This study has determined that PWD and P_-max_, which are both risk factors for atrial arrhythmias, are significantly reduced within 12^th^ months after successful CTO PCI regardless of the target vessel.

## Introduction

In patients with paroxysmal atrial fibrillation (AF), the duration of the P-wave appears to be essential to assess the inhomogeneity of electrical atrial activity. Additionally, P-wave dispersion (PWD) obtained from the standard 12-lead electrocardiography (ECG) is considered to reflect the homogeneity of the atrial electrical activity, and it appears to be quite promising in the perspective of AF prediction.^1^ A prolonged duration of PWD has been reported to increase the risk of developing AF in cases without underlying heart disease.^2^ AF is the commonly observed cardiac arrhythmia, with a 25% lifetime risk along with associated complications in the population, including heart failure, stroke, and death.^3^ In developed countries, hypertension and coronary artery disease (CAD) are the main clinical diagnoses associated with AF. CAD rarely causes direct atrial ischemia and AF. More frequently, CAD causes severe ventricular ischemia leading an increase in intra-atrial pressure and AF.^4^

Chronic total occlusion (CTO) is commonly observed in daily practice, and it is detected roughly 20% of all cases undergoing coronary angiography (CAG).^5^ The frequency of CTO in cases with known CAD is between 30% and 50%.^6^ Some studies have investigated whether CTO percutaneous coronary intervention (PCI) can improve ventricular repolarization homogeneity. For example, Erdogan et al showed that after CTO PCI, effective revascularization may result in improved regional heterogeneity of myocardial repolarization, as demonstrated by lower QT dispersion (QTd).^7^ In addition, there is existing data on P-wave maximum time (P_-max_) and PWD showing an association with myocardial ischemia.^8^ However, the data is lacking on the outcome of CTO PCI revascularization on P_-max_ and PWD parameters in the ECG and their main effects on atrial conduction. Therefore, we aimed to appraise the effect of CTO PCI revascularization on atrial conduction abnormalities, including P_-max_ and PWD, in patients before and after procedure at 12 months.

## Materials and Methods

### Study population

This study was planned as a retrospective and cohort study, and it was carried out conforming to the principles outlined in the Declaration of Helsinki. Initially, 108 consecutive CTO cases who underwent PCI at Istanbul Haseki Training and Research hospital between January 2014 and January 2020 were analyzed. Patients who had AF, atrial flutter and pre-excitation and those cases with permanent pacemakers, severe electrolyte imbalance before and after CTO procedure, undergoing hemodialysis, having congenital heart disease were not excluded. Additionally, patients whose ECG records were unsuitable for analyses and those with missed follow-up were excluded from the study group (n = 18). The hospital’s electronic medical database systems provided demographic, clinical, echocardiographic and biochemical data for all patients. All cardiovascular risk factors were noted. In the present investigation, each case was treated with acetylsalicylic acid, beta blocker, angiotensinogen converting enzyme inhibitors/angiotensinogen receptor blockers, and statin therapy unless contraindicated. Blood samples were taken from the antecubital vein at our hospital within 24 hours of admission. An autoanalyzer was used to quantify total white blood cell (WBCs) count, hemoglobin, platelets, neutrophils, and lymphocytes. An automated chemistry analyzer was used to assess the plasma levels of fasting blood glucose, total cholesterol, triglycerides, high-density lipoprotein cholesterol, low-density lipoprotein cholesterol, and creatinine in all patients. A Roche Diagnostics Cobas 8000 c502 analyzer was used to test C-reactive protein (CRP) levels (Roche Holding AG, Basel, Switzerland).

### CTO procedure

A CTO lesion, under the guidance of the Euro CTO Club, was defined as a completely occluded lesion for more than 3 months.^9^ CTO lesions were also classified according to coronary collateral flow using the Rentrop classification. Rentrop classification defines collateral circulation between 0 and 3. Rentrop 0: no visible collateral flow, Rentrop 1: filling the lateral branches of the occluded artery, Rentrop 2: partial filling of the epicardial coronary vessel, Rentrop 3: complete collateral filling of the epicardial coronary artery.^10^ Recanalization of the CTO indication was confirmed with the presence of angina pectoris in the occluded artery area and/or myocardium (viable or ischemic) with diagnostic tools. After stent deployment, a successful CTO PCI was defined as restoring grade 3 Thrombolysis in Myocardial Infarction (TIMI) flow with less than 20% remaining stenosis in the CTO segment.

### ECG analysis

**Figure 1:**
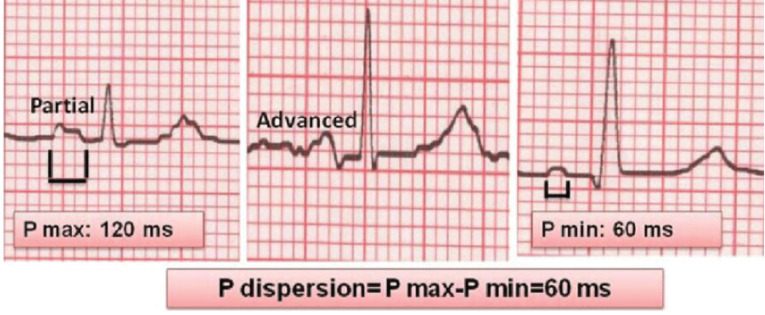
An example of measurement of P wave maximum, P wave minimum, and P wave dispersion

A conventional 12-lead ECG (Schiller Cardiovit AT-102-G2 machine) with 25 mV recording was acquired after a 10-minute rest in the supine position for each case included in the study. In all cases, baseline ECG and 12^th^ months ECG were obtained. The P wave was estimated from the first evidence of upward departure from the baseline until the point of return to the baseline. The difference between the P-wave maximum time (P_-max_), and P-wave minimum time (P_-min_) observed in any of the 12 leads was termed as PWD (Figure 1). For each lead, P-wave duration was calculated manually on a high-resolution computer screen by two investigators who were blinded to the clinical details of the cases. All P-wave parameters were measured by two cardiologists separately before and one year after the procedure, and the mean of these two values ​​were accepted as the PWD, P_-max_, and P_-min_. Intra-and inter-observer coefficients of variation were 3% and 4% for P wave duration, and 4% and 5% for PWD, respectively.

### Statistical analysis

The statistical analysis was performed using SPSS version 15.0 (IBM Corporation, USA) for Windows (Microsoft Inc, USA). Mean, standard deviation (SD), median, and [interquartile range (IQR)] or frequencies were used to convey descriptive data. The Mann-Whitney U test or the independent samples Student’s t test were used to compare group means. The Pearson’s 2 test was used to compare categorical variables. Statistical significance was defined as a *P* <  0.05.

## Results

In overall, 90 consecutive CTO cases were analyzed. The study cohort was categorized into two groups as successful CTO PCI (n = 64; 71%) and unsuccessful CTO PCI groups (n = 26; 29%).

Pre-procedural demographic, clinical, laboratory, angiographic data are displayed in Table 1. Both groups, except for age and hypertension, were similar in terms of demographic and clinical aspects. Additionally, successful revascularization of CTO vessel was significantly lower in cases with previous coronary aorto bypass grafting. In regard to laboratory data, CRP levels were significantly elevated and hemoglobin levels were significantly reduced in the failed PCI group. CTO segment was mostly located in the right coronary artery (RCA; 55.6%). Afterwards, the left anterior descending artery (LAD) and circumflex artery (CX) were target arteries in 23.3% and 21.1% of the patients, respectively.

**Table 1:** Demographic, clinical, laboratory and electrocardiographic parameters of the study cohort

	**All population** **(n=90)**	**Successful CTO PCI** **(n=64)**	**Unsuccessful CTO PCI** **(n=26)**	***P*** ** value**
Male gender, n %	57 (63.3)	36 (56.3)	21 (80.8)	0.03
Age, years	56.0 ± 9.2	53.8 ± 8.6	61.6 ± 8.4	< 0.01
Hypertension, n (%)	54 (60)	32 (50)	22 (84.6)	< 0.01
Diabetes mellitus, n (%)	57 (64)	39 (61.9)	18 (69.2)	0.51
Smoking, n (%)	38 (42.2)	26 (40.6)	12 (46.2)	0.63
Previous MI, n (%)	34 (37.8)	23 (35.9)	11 (42.3)	0.57
Previous CABG, n (%)	18 (20)	9 (14.1)	3 (34.6)	0.03
Previous CVA, n (%)	2 (2.2)	2 (3.1)	0 (0)	0.36
LVEF, %	55.7 ± 3.9	55.3 ± 3.9	56.5 ± 3.9	0.18
Creatinine, mg/dl	0.8 [0.8-1.0]	0.8 [0.8-0.9]	0.8 [0.8-1.0]	0.53
TC, mg/dL	204.4 ± 36.7	208.7 ± 39.1	193.8 ± 28	0.08
LDL-C, mg/dL	134.6 ± 32.9	143.8 ± 35.2	135.9 ± 26.3	0.30
HDL-C, mg/dL	41.7 ± 6.6	41.6 ± 7.0	41.9 ± 5.7	0.84
CRP, mg/dl	4.9 [1.5-12]	4.4 [1.3-8.3]	12.8 [1.7-14.0]	< 0.01
Uric acid, mg/dl	5.4 ± 1.8	5.5 ± 1.9	5.2 ± 1.4	0.57
Hemoglobin g/dl	13.8 ± 2.1	14.2 ± 1.7	12.7 ± 2.8	< 0.01
Neutrophil, µx10^3^ /µL	4.9 ± 1.3	5.1 ± 1.4	4.5 ± 1.2	0.08
Lymphocyte, µx10^3^ /µL	1.5 ± 0.4	1.4 ± 0.39	1.6 ± 0.4	< 0.01
Platelet, µx10^3^ /µL	358 ± 59	380 ± 44	304 ± 57	< 0.01
Target vessel, n (%)				
LAD	21 (23.3)	12 (18.8)	9 (34.6)	
Cx	19 (21.1)	11 (17.2)	8 (30.8)	0.04
RCA	50 (55.6)	41(64.1)	9 (34.6)	
Rentrop classification, n (%)				
Rentrop 1	22 (24.4)	12 (18.8)	10 (38.8)	0.05
Rentrop 2	35 (38.9)	23 (35.9)	12 (46.2)	0.37
Rentrop 3	33 (36.7)	29 (45.3)	4 (15.4)	< 0.01

Abbreviations: LVEF, left ventricular ejection fraction; MI, myocardial infarction; CABG, coronary artery by-pass grafting; CVA, cerebrovascular accident; TC, total cholesterol; LDL-C, low density lipoprotein cholesterol; HDL-C, high density lipoprotein cholesterol; CRP, C-reactive protein; RCA, right coronary artery; Cx, circumflex artery, LAD, left anterior descending artery

Pre-CTO PCI P-wave parameters and post-CTO PCI P-wave parameters at 12^th^ months are summarized in Table 2. At baseline, P-wave parameters, including P_-max_, P_-min_, and PWD, were similar between two groups. P_-max_ values at 12^th^ months after successful CTO PCI were statistically lower than the baseline (114.8 ± 3.3 ms vs 108.8 ± 3.3 ms, *P* <  0.001, respectively). P_-min_ values at 12^th^ months were statistically higher than the baseline (56 ± 3.3 ms vs 57.2 ± 4.5 ms, *P* = 0.002, respectively). PWD was significantly lower than the baseline at 12^th^ months (58.7 ± 6.1 ms vs 51.4 ± 6.3, *P* <  0.001, respectively). In contrast to that, in the failed CTO PCI group, no significant changes were detected at 12^th^ months in terms of P_-max_, P_-min_, and PWD values.

**Table 2:** Comparison of pre- and post-PCI P-wave parameters of all cases

	**Pre-PCI**	**Post-PCI at 12** ^th^ ** moths**	***P*** ** value**
Successful PCI group			
P_-min_, ms	56 ± 3.3	57.2 ± 4.5	0.02
P_-max_, ms	114.8 ± 3.3	108.8 ± 3.3	< 0.01
PWD, ms	58.7 ± 6.1	51.4 ± 6.3	< 0.01
Unsuccessful PCI group			
P_-min_, ms	56.4 ± 2.2	56.7 ± 2.4	0.16
P_-max_, ms	114.4 ± 2.2	114.2 ± 2.3	0.33
PWD, ms	56.7 ± 2.4	57.5 ± 4.7	0.33

Abbreviations: PCI, percutaneous coronary intervention; P_-max_, P-wave maximum time; P_-min_, P-wave minimum time; PWD, P wave dispersion

When the successful PCI group was evaluated based on the Rentrop classification, it was noted that they were mostly Rentrop class 3 patients (45.3%). Compared to pre-CTO PCI values in all Rentrop classes at the 12^th^ months, PWD values were most reduced in Rentrop class 3 patients (Table 3).

**Table 3:** Comparison of pre- and post-PCI electrocardiographic parameters based on the Rentrop classification

	**Pre-PCI**	**Post-PCI at 12** ^th^ ** months**	***P*** ** value**
Rentrop 1			
P_-min_, ms	55.1 ± 3.5	57.6 ± 5.3	0.05
P_-max_, ms	116.2 ± 3.0	112.5 ± 3.0	< 0.01
PWD, ms	60.2 ± 6.6	55 ± 7.2	< 0.01
Rentrop 2			
P_-min_, ms	55.9 ± 2.8	55.9 ± 3.1	1.0
P_-max_, ms	114.6 ± 2.8	110.7 ± 4.0	< 0.01
PWD, ms	58.4 ± 5.5	54.1 ± 5.4	< 0.01
Rentrop 3			
P_-min_, ms	57 ± 2.8	58 ± 3.7	0.02
P_-max_, ms	113.8 ± 2.8	108.6 ± 3.6	< 0.01
PWD, ms	56.7 ± 5.5	51.1 ± 6.6	< 0.01

Abbreviations: PCI, percutaneous coronary intervention; P_-max_, P-wave maximum time; P_-min_, P-wave minimum time; PWD, P wave dispersion

## Discussion

Our study revealed that P wave duration and PWD were significantly reduced after revascularization with PCI in patients with CTO at 12^th^ months follow-up. We assume that atrial conduction disturbance might be improved by CTO PCI revascularization, thereby possibly reducing atrial arrhythmias triggered by pro-arrhythmic effects of CTO.

The identification of atrial fibrosis in the heart failure experimental model at the beginning of the century and, consequently, causing the slowing of conduction and increased conduction heterogeneity, it was accepted that atrial structural remodeling contributed to the continuation of AF.^11-13^ Experimental and clinical studies have showed various AF risk factors, including heart failure, valvular heart disease, and endurance training.^11-15^

PWDs are simple and inexpensive measurements reflecting the regional heterogeneity of atrial repolarization. The PWD is an index that reflects the risk of AF.^16^ PWD changes have been studied in patients with acute myocardial infarction, chronic coronary syndrome, and angioplasty-induced myocardial ischemia. In a study of Dilaveris et al, PWD was found to be longer in cases with stable anginal episodes compared with those who did not have.^17^

The CTO of the coronary arteries is detected in 35-50% of cases with significant CAD undergoing diagnostic CAG.^18^ Remarkably, PCI has become a widely accepted treatment strategy for CTO in current practice.^19^ Studies found that improved left ventricular (LV) systolic function, less anginal symptoms, greater exercise capacity, decreased need for CABG, and, most significantly, longer survival rates had all been linked to successful PCI of CTO.^20,21^ The effect of revascularization on outcome in cases with LV systolic dysfunction has recently been answered by the results of the randomized Surgical Treatment for Ischemic Heart Failure(STICH) study.^22^ PCI has been reported to benefit for CTO cases by reducing ischemic symptoms and mortality owing to the improvement of myocardial function.^23,24^

The observations of our study showed that PWD was significantly reduced after revascularization of CTO. We consider that some mechanisms may be the underlying reasons for the improvement of atrial conduction.^23^ LV diastolic and/or systolic functions may be reduced in CTO patients due to prolonged ischemia and injury. An increase in left atrial pressure and diameter is expected to develop as a consequence of elevated in LV end-diastolic pressure (LVEDP). Conduction abnormalities can occur in the atrial myocardium as a consequence of increased left atrial diameter and pressure. Improvement in atrial electrical conduction is expected as a result of a reduction in LVEDP after CTO recanalization.^24^ In our study, we observed that PWD was significantly reduced, which might mean an improvement in the progression of sinus impulses. In CTO conditions, a direct atrial wall ischemia can occur as a result of atrial fibrosis, which may be another explanation for the prolongation of PWD. As a result of fibrosis in the atrial wall, there may be no homogeneity in atrial conduction. P_-max_ and PWD were significantly reduced in cases with CTO lesions after recanalization, and it may be due to the healing of atrial tissue as well as improvement of atrial conduction as noted above.

Even though successful CTO recanalization has been associated with better clinical benefits, the outcomes of patients with AF undergoing CTO PCI have not been investigated yet. The relationship between PWD and AF had been shown in a previous study.^25^ However, we did not show that CTO PCI directly reduced the risk of AF in our study; however, since the relationship between PWD and AF was well-known, we thought that a decrease in PWD might decrease the incidence of AF. Besides that, we found that P_-max_ and PWD were significantly reduced after recanalization in patients with CTO regardless of vessel type.

Several limitations are evident in our study. The main limitation was retrospective design of the study. The sample size was also relatively small; hence, further prospective studies with larger cohorts may be needed to confirm the results. Although patients who developed AF following CTO PCI procedure were excluded, a 24-hours Holter monitoring was not performed to detect AF during long-term follow-up. Lastly, we measured the conduction times only with ECG and we did not perform electrophysiological study, which is the gold standard method that should be used to validate our results.

## Conclusion

The present investigation has showed that P-_max_ and PWD might be significantly reduced at 12^th^ months following successful CTO recanalization by PCI.

## Acknowledgements

None.

## Competing interest

The authors state that they have no competing interests in the publication of this paper.

## Ethical approval

The Local Ethic Committee examined and approved the study protocol (Decision number: 276).

## Funding

None.
